# Full-Length Genome of the Equine Influenza A Virus Subtype H3N8 from 2019 Outbreak in Saudi Arabia

**DOI:** 10.3390/ani12192720

**Published:** 2022-10-10

**Authors:** Fanan A. Alaql, Ali N. Alhafufi, Samy Kasem, Yousef M. O. Alhammad, Hassan Albaqshi, Ameen Alyousaf, Faisal M. Alsubaie, Ahmed N. Alghamdi, Ahmed S. Abdel-Moneim, Sulaiman A. Alharbi

**Affiliations:** 1 Virology and Genome Department in Central Veterinary Laboratory (CVL), Ministry of Environment, Water and Agriculture (MEWA), P.O. Box 15831, Riyadh 11454, Saudi Arabi; 2 Botany & Microbiology Department, College of Science, King Saud University, Riyadh 12372, Saudi Arabia; 3 Department of Virology, Faculty of Veterinary Medicine, Kafrelsheikh University, El Geish Street, Kafrelsheikh 33516, Egypt; 4 Department of Microbiology, College of Medicine, Taif University, Taif 21944, Saudi Arabia

**Keywords:** equine influenza, H3N8, phylogenetic analysis, Saudi Arabia, EIV vaccine

## Abstract

**Simple Summary:**

Equine influenza is a highly contagious respiratory viral disease. The current study is the first to provide a description of the full-length genome sequence and surveillance of recent exposure to the equine influenza virus (EIV) during the 2019 epidemic in Saudi Arabia. This epidemic was benign, since it resulted in low case fatality (0.45%, 1/224). The viruses detected in the current study were found to be related to subtype H73N8 clade 1 of the Florida sublineage. Full-length genome sequencing revealed no evidence of major genetic changes or of reassortment among the eight segments of the viral genome. However, the Saudi strains showed a considerable number of amino acid substitutions in the signal peptide (2 amino acid substitutions), HA1 (10 amino acid substitutions) and HA2 (4 amino acid substitutions) in the haemagglutinin glycoprotein in comparison to clade 1 Florida sublineage vaccinal strains. These findings should be considered during selection of the equine influenza vaccine strains approved for use in Saudi Arabia.

**Abstract:**

Equine influenza is a major cause of respiratory infections in horses and can spread rapidly despite the availability of commercial vaccines. This study aimed to screen the incidence of equine influenza virus (EIV) and molecularly characterize the haemagglutinin and neuraminidase from positive EIV field samples collected from Saudi Arabia. Six-hundred twenty-one horses from 57 horse barns were screened for the presence of the clinical signs, suggestive for equine influenza, from different parts of Saudi Arabia. Nasopharyngeal swabs were collected from each horse showing respiratory distress. Samples from the same horse barn were pooled together and screened for the presence of the influenza A virus using quantitative real time reverse transcriptase polymerase chain reaction (qRT-PCR). Selective positive samples were subjected to full-length genome sequencing using MiSeq Illumina. Out of the total 57 pools, 39 were found positive to EIV using qRT-PCR. Full-length gene sequences were compared with representative EIV strains selected from the GenBank database. Phylogenetic analysis of the HA and NA genes revealed that the identified virus strains belong to H3N8 clade 1 of the Florida sublineage and were very similar to viruses identified in USA in 2019, with no current evidence for reassortment. This is one of the first reports providing detailed description and characterization of EIVs in Saudi Arabia. Detailed surveillance and genetic information sharing could allow genetic evolution of equine influenza viruses to be monitored more effectively on a global basis and aid in refinement of vaccine strain selection for EIV.

## 1. Introduction

Equine influenza (EI) is an acute, contagious infectious disease of horses, donkeys, and other members of the family *Equidae* caused by the equine influenza A virus of the genus *Orthomyxovirus* within the Family *Orthomyxoviridae* [1]. The characteristic clinical signs of influenza virus infection in equines include high fever, cough, and serous nasal discharge, and abortion may occur in pregnant animals. The signs could precede to pneumonia, enteritis, emphysema and even death in untreated cases [2].

The World Organization for Animal Health (WOAH) classifies the equine influenza virus among the most important notifiable diseases. The epidemic of EIV is extremely strong; once established, it rapidly spreads to whole equine populations in a region. The mode of transmission is mainly through direct contact with infected animals or indirectly through people, infected fomites, or vehicles. Horse transport, especially cross-border transportation in the case of horse races, is the main reason for the spread of equine influenza from one country to another [3,4].

Influenza A viruses contain single-stranded negative-sense segmented genome (8-segments) that includes PB2, PB1, PA, HA, NP, NA, M, and NS segments. Based on both hemagglutinin (HA) and neuraminidase (NA) surface glycoproteins, influenza A viruses are subtyped into dozens of subtypes. To date, 18 types of HA and 11 types of NA exist, with more than 130 subtype combinations found in nature [5]. However, equine influenza is known to be caused by two main subtypes: H7N7 and H3N8 [6]. Additionally, avian influenza subtype H5N1 was reported to cause respiratory distress in equines [7]. H7N7 (A/equine/Prague/1/56) was firstly isolated in Czechoslovakia in 1956, while in 1963, H3N8 (A/equine/Miami/I/63) was first detected in the USA from animals imported from Argentina [8]. The latter subtype spread globally, while the former has not been detected in equine populations since the late 1970s [9]. American and Eurasian lineages of the H3N8 were detected in the 1980s [10,11]. Florida sublineage clades 1 and 2, South American and Kentucky, have emerged as descendants of the American lineage [12,13]. The Florida clade 1 sublineage circulated in North America, while the Florida clade 2 sublineage circulated in Europe [13]; however, both are currently circulating worldwide [14].

In Saudi Arabia, there is limited information about the EIV. A single report confirmed the presence seropositive equine sera against H3 using a hemagglutination inhibition test, but no virus was detected using qRT-PCR in the examined swabs of the horses suffering from respiratory disease in Western Saudi Arabia. The second was conducted among horses in Eastern and Central Saudi Arabia using molecular (qRT-PCR) and serological (ELISA and HI tests) surveillance for EIV H3N8. The results showed that none of the swabs had detectable influenza A virus RNA with qRT-PCR, while 81 out of 145 sera samples tested by ELISA (55.9%) were positive for equine H3, and 98 (67.6%) of 145 sera tested by HI tests were positive [15]. The current study aimed to screen the presence of viral nucleic acid using qRT-PCR as an indicator of recent infection and characterize the full-length genome from the positive samples in different regions of Saudi Arabia during the outbreak of equine influenza in January 2019.

## 2. Materials and Methods

### 2.1. Sample Collection

The first clinical cases of equine influenza-like symptoms were observed in the Alganderia region in Riyadh Province in the central region of Saudi Arabia in January 2019. A surveillance team from the Ministry of Environment, Water and Agriculture (MEWA) investigated the affected horses, especially around the focal point of disease. All the examined horses were related to the Arabian horse breed. The most important clinical features were the presence of the progression of severe respiratory clinical signs in most horses. The signs included fever and dry cough followed by serous nasal discharge, submandibular lymphadenopathy, weakness, and anorexia.

Control measures for EIV including restriction of animal movement, isolation, and treatment of infected animals; hygienic disposal of animal waste was implemented. After confirmation of suspected cases, all MEWA branches in Saudi Arabia were notified of the EIV cases. In return, the MEWA instructed all field veterinarians across the entire country to report any suspected cases and to collect samples from these cases, to be sent to the Central Veterinary Laboratory (CVL), (MEWA) for diagnosis and sequencing. Field veterinarians visited horse barns with affected animals daily until the complete cessation of disease or death of the animals. Cases of presumptive EIV were reported in other regions, namely Riyadh, Eastern region, Al-Qassim, Makkah, Al-Madinah, and Tabuk. Swab samples were collected from each horse showing respiratory distress. Each swab was placed in 3 mL of viral transport medium UTM^®^ USA.

### 2.2. Real-Time PCR Detection of EIV Sequencing

Viral RNA nucleic acid was extracted from 200 µL of the nasal swab pool (50 µL of each sample pooled into a single pool) by high pure viral RNA kit (ROCHE, Indianapolis, IN, USA). The viral RNA genome was eluted and quantified by the Nanodrop and Qubit quantification apparatus. A rapid detection of EIV from the suspected cases was done by TaqMan quantitative reverse transcriptase real-time polymerase chain reaction (qRT-PCR) assay targeting the Influenza A matrix (M) gene using M primers: (M Forward Primer: 5′-AGA TGA GTC TTC TAA CCG AGG TCG-3′; M Reverse Primer: 5′-TGC AAA AAC ATC TTC AAG TCT CTG-3′; M Probe: 5′-[FAM]-TCA GGC CCC CTC AAA GCC GA-[TAMRA]-3′) [16,17]. The qRT-PCR assay was conducted in a 25 µL reaction mixture containing 12.5 µL of 2 × RT-PCR buffer (Thermo Scientific, Waltham, MA, USA), 1 µL of 25 × RT-PCR enzyme mix, 1 µL (0.4 µmol) each of forward and reverse primers (10 µmol/L), 1 µL (0.24 µmol) of probe (6 µmol/L), 6 µL of nuclease-free water and 2.5 µL of extracted RNA. The reaction was done on a Light Cycler 2.0 PCR instrument (Roche Diagnostics, Penzberg, Germany).

### 2.3. Full-Length Genome Sequencing

The full-length genome of samples showed high positive results from qRT-PCR. Common universal primers were used to detect all eight segments in a single reaction: uni12G (55′′-GCC GGA GCT CTG CAG ATA TCA GCG AAA GCA GG-33′′), Uni-12R (55′′GCC GGA GCT CTG CAG ATA TCA GCR AAA GCA GG-33′′), and common Uni-13 (55′′-CAG GAA ACA GCT ATG ACA GTA GAA ACA AGG-33′′), as previously described [18]. Two RT-PCR reactions were performed. Both common-uni12R and common-uni13 primers were used in the first reaction, while common-uni12G and common-uni13 primers were used in the second RT-PCR reaction. The latter was intended to improve the amplification of the replication genomic segments of IAV: PB2, PB1, and PA. The One-Step RT-PCR High Fidelity kit containing SuperScript™ III (Invitrogen) was used. A total of 5.0 μL of the extracted RNA from each pool as the target of amplification and 0.2 μM of each primer were used. Reverse transcription was conducted for 30 min at 50 °C, followed by 2 min pre-denaturation at 94 °C. This step was followed by 34 cycles of denaturation at 94 °C for 15 s, annealing at 50 °C for 30 s, and extension at 68 °C for 3 min, repeated for 34 cycles. At the end, there was an additional single elongation cycle at 72 °C for 5 min. PCR was first analyzed using 1% gel electrophoresis. Equal volumes of both RT-PCR reactions were pooled and used for the obtaining the full-length genome of the EIV. The PCR product was first subjected to purification using cleanup (AMPure XP, Beckman Coulter, Inc., Kraemer Blvd. Brea, CA 92821 USA), quantification by Qubit highly sensitive DNA kit USA, qualification by bioanalyzer (High sensitive DNA assay kit, USA) prior to loading into the MiSeq reagent kit v2 (2 × 250 bp). All steps were conducted according to illumina manufacturing in the Genome Department, Central Veterinary Diagnostic Laboratory, Ministry of Environment, Water and Agriculture, Riyadh, Saudi Arabia.

### 2.4. Sequence Analysis

Nucleotide sequences were trimmed by removal of the primer sequences prior to Blastn analysis. All sequences were deposited into GenBank (Accession numbers for PB2: (MW255293, MW261771, MW261751); PB1: (MW255294, MW261752, MW261772); PA: (MW255295, MW261773, MW261753); HA: (MW255300, MW261778, MW261758); NP: (MW255296, MW261774, MW261754); NA: (MW255298, MW261776, MW261756); M1: (MW255299, MW261777, MW261757); NS: (MW255297, MW261775, MW261755)).

Phylogenetic analyses for the PB2, PB1, PA, HA, NP, NA, M, and NS nucleotide sequences and deduced amino acid alignments were performed using MEGA 5.2 [19]. The phylogenetic trees were performed using the Maximum Likelihood Heuristic Method and the Tamura-Nei model with the Nearest-Neighbor-Interchange (NNI). The percentage of replicate trees in which the associated taxa clustered together are shown next to the branches [20]. The initial tree(s) for the heuristic search were obtained automatically by applying Neighbor-Join and BioNJ algorithms to a matrix of pairwise distances estimated using the Tamura-Nei model and then selecting the topology with superior log likelihood value.

## 3. Results

### 3.1. Morbidity and Mortality of EIV

Horses with typical clinical signs of EIV were reported in the Eastern Regions, Riyadh, Al-Qassim, Makkah, Al-Madinah, and Tabuk (Table 1). Affected horses showed fever, dry cough followed by serous nasal discharge, submandibular lymphadenopathy, weakness, and anorexia.

The veterinary clinical unit received cases of infected horses, with a total number of *n* = 224, from different regions. Nasal swabs were collected from affected horses. From each horse barn, a pool of samples was conducted with a total of 57 pools.

Epidemiological analysis indicated that a considerable number of examined horses 224/621 (36.1%) showed clinical disease suggestive for equine influenza. The outbreak showed low mortality rate, since only one horse died (Table 1).

### 3.2. Real-Time PCR and Phylogenetic Analysis

Thirty-nine out of fifty-seven of the examined nasal swabs collected from the affected horses were found positive for EIV RNA using qRT-PCR. Three out of the thirty-nine rRT-qPRC positive samples that showed the highest viral RNA concentrations (*Ct* 24 to 26) were used for full-length genome sequencing of the virus using next generation sequencing. The nucleotide sequences form the Saudi stains were processed and then compared the with 35 EIV H3N8 HA sequences related to main different clades, including the original viruses that are currently used as vaccines against EIV as well as closely related strains in the blast search. Matched genes from the selected strains were also used to compare the remaining seven genes (PB2, PB1, PA, NP, NA, M, NS) for the same selected strains. Accordingly, individual phylogenetic trees were obtained for each of the eight segments.

A/Equus ferus caballus/USA/51053/2019 was found to be the closely related strain. For all gene segments, the trees contain different clusters of American (American, Florida 1, and Florida 2) and Eurasian lineages. The different genes of the Saudi strains of the equine influenza subtype H3N8 all belonged to Florida clade 1.

**Figure 1:**
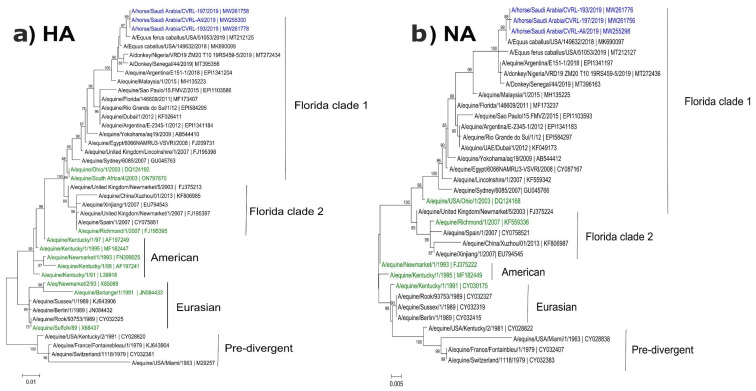
Phylogenetic analysis of haemagglutinin and neuraminidase genes of Saudi EIV subtype H3N8 strains in comparison to representative published sequence. (**a**) Phylogeny of the hemagglutinin (HA) gene. (**b**) Phylogeny of the neuraminidase (NA). Phylogenetic trees were created using the maximum likelihood method with 1000 bootstraps. Saudi strains are presented in blue. Virus strains used for vaccine production are presented in green.

**Figure 2:**
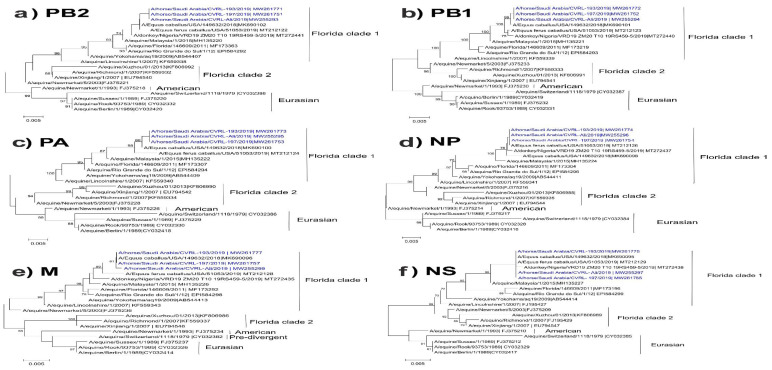
Phylogenetic tree of influenza virus polymerase complex (PB2, PB1, PA), nucleoprotein (NP), matrix protein (M), and non-structural gene (NS) nucleotide sequences of the Saudi H3N8 strains in comparison to representative published sequences. (**a**) ML phylogenetic tree of PB2 gene, (**b**) ML phylogenetic tree of PB1 gene, (**c**) ML phylogenetic tree of PA gene, (**d**) ML phylogenetic tree of NP, (**e**) ML phylogenetic tree of M gene, and (**f**) ML phylogenetic tree of NS gene. Phylogenetic trees were created using the maximum likelihood method with 1000 bootstraps. Saudi strains are presented in blue.

For the HA and NA trees, the pre-divergent strains, including the original A/equine/USA Miami/63, were clustered together. Within the same clade, virus strains from the same year were closely related to each other. Saudi strains were found to be related to Florida clade 1 and closely related to strains from USA, Senegal, Nigeria, and Malaysia (Figure 1 and Figure 2). In general, nucleotide sequences of the different genes displayed similar tree patterns, with no evidence of reassortment among the Saudi strains (Figure 1 and Figure 2).

### 3.3. Amino Acid Variations

**Figure 3:**
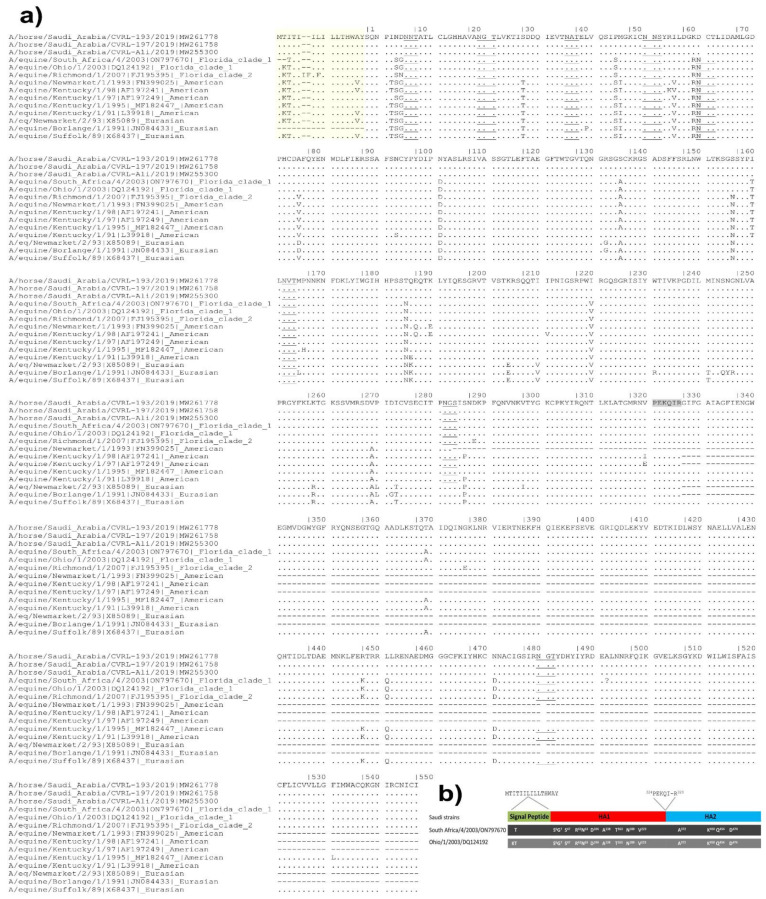
Deduced amino acid sequence of the hemagglutinin glycoprotein of the Saudi H3N8 equine influenza virus strains in comparison to the virus strains used for the production of H3N8 vaccines. (**a**) Deduced amino acid sequences. Dots mean identical sequences. Signal peptide is marked by yellow boxing. Polybasic cleavage site is highlighted in grey. N-glycosylated motives (NXT or NXS, except X = P) are underlined. (**b**) A schematic representation of the HA protein of Saudi strains compared to the reference strains Ohio and South Africa California clade 1 vaccine strains.

We analyzed the amino acid differences in HA glycoprotein between the Saudi strains and between the Saudi H3N8 strain and the original viruses from which the currently used commercial EIV vaccines were developed The commercially available vaccines include Florida clade 1 vaccines (A/equine/Ohio/1/2003 and A/equine/South Africa/4/2003), Florida clade 2 vaccine (A/equine/Richmond/1/2007), the American clade vaccines (A/equine/Kentucky/1/91, A/equine/Kentucky/1/95, A/equine/Kentucky/1/97, A/equine/Kentucky/1/98, and A/equine/Newmarket/1/1993) as well as Eurasian clade vaccines (A/equine/Suffolk/89, A/equine/Borlange/1/1991, and A/equine/Newmarket/2/1993) (Figure 1 and Figure 3).

The HA of the Saudi strains are identical to each other and showed only two amino acid substitutions form A/Equus ferus caballus/USA/51053/2019 at T163I and A372T (Table 2). The Saudi strains contain seven N glycosylation sites, while all the vaccinal strains contain an additional N glycosylation site at the position 63. The first 15 amino acid signal peptide, MTITIILILLTHWAY, although conserved among the Saudi, showed various amino acid substitutions in different original viruses that are currently used as vaccinal strains. Two T to K and three I to T amino acid substitutions (signal peptide numbering) were detected in A/equine/Kentucky/1/95| MF182447 and A/equine/Kentucky/1/97|AF197249. Two T to K, three I to T and 14 A to V amino acid substitutions (signal peptide numbering) were detected in A/equine/Suffolk/89|X68437, A/equine/Newmarket/1/1993| FN399025, and A/equine/Kentucky/1/91| L39918 as well as A/equine/Kentucky/1/98|AF197241. Two T to K, three I to T, and seven L to F amino acid substitutions (signal peptide numbering) as well as two amino acid insertions (FI) were detected in A/equine/Richmond/1/2007|FJ195395. Two T to K and three I to T amino acid substitutions (signal peptide numbering) were detected in the A/equine/Ohio/1/2003| DQ124192. Three I to T amino acid substitutions (signal peptide numbering) were detected in A/equine/South Africa/4/2003 |ON797670. No variation in the HA cleavage site, ^324^PEKQI-R^329^, was found among all the examined Saudi and vaccinal strains (Figure 3).

Both A/equine/Ohio/1/2003 and the A/equine/South Africa/4/2003 showed 10 amino acid substitutions in the HA1 (N6S, D7G, P47S, K62R, D63N, N104D, S138A, I163T, T188N, I2003V) and three in HA2 (R450K, L454Q, and N474D). A/equine/Richmond/1/2007 showed 12 amino acid substitutions in the HA1 (N6S, D7N, P47S, K62R, D63N, A78V, N104D, S138A, S159N, T188N, I2003V, D291E) and five in HA2 (A372T, G379E, R450K, L454Q and N474D). Other vaccinal strains that belong to other clades showed a higher number of amino acid substitutions, including A/equine-2/Kentucky/97 (16 amino acid substitution in the HA1), A/equine/Kentucky/1/91 (18 amino acid substitutions in the HA1 and 3 in HA2), A/equine/Newmarket/1/1993 (18 amino acid substitutions in the HA1), A/Newmarket/2/93-Eurasian (20 amino acid substitutions in the HA1), A/equine/Kentucky/1/98 (22 amino acid substitutions in the HA1), A/equine/Suffolk/89 (22 amino acid substitutions in the HA1 and 3 in HA2) and A/equine/Borlange/1/1991 (28 amino acid substitutions in the HA1 and 3 in HA2) (Figure 3).

The deduced amino acid sequences of different genes the Saudi strains were compared to A/Equus ferus caballus/USA/51053/2019 and other closely related strains. The NA gene of the A/horse/Saudi Arabia/CVRL-197/2019| MW261756 showed a single silent point mutation at the position of 681 G to A (data not shown). PB2 of A/horse/Saudi Arabia/CVRL-193/2019 showed H27Q, A/horse/Saudi Arabia/CVRL-Ali/2019 showed K 482T, while A/horse/Saudi Arabia/CVRL-197/2019 showed K2R amino acid substitution in the NP. All Saudi strains showed the following amino acid substitutions: I149V in the PB1 I90V and I460M in the PA, T163I and A372T in the HA, as well as I74M and D75E in the NA (Table 2).

## 4. Discussion

In our study, 224 out of the 621 examined horses were found clinically ill. A total of 39/57 pool samples were found positive to IAV. Three samples, with Ct ranging from 24–26, were subjected to next generation sequencing, and full-length genomes of H3N8 were obtained. In Saudi Arabia, three were previously reported in EIV; the first one, reporting on 100 animals showing influenza symptoms during 2009–2010, was examined by qRT-PCR. However, negative results were obtained from all the examined horse samples (n: 100) [21]. The second report examined 145 sera, 323 nasal, and 323 rectal swab samples from horses. The sera were tested by ELISA assays and by hemagglutination inhibition (HI) tests, while the swabs were tested by qRT-PCR assay. Negative results were also obtained from all the examined swabs. Out of the 145 tested serum samples, 81 (55.9%) were positive by ELISA and 98 (67.6%) tested positive by HI using equine H3 hemagglutinin [15]. The last report detected EIV using real-time PCR results in 35.1% of the tested horses, donkeys, and ponies but failed to characterize the virus [22].

EIV subtype H3N8 has previously been reported in Middle Eastern countries, such as Morocco in 1997 and 2004 [23], Tunisia in 1998 [24], Egypt in 2000 [25], Algeria in 2011 [26], and Dubai in 2012 [27]. In addition, there are GenBank submissions of EIV genes from Egypt (2008 and 2018) but no associated publications describing outbreaks.

The phylogenetic tree of the Saudi H3N8 subtype revealed that it belongs to Florida clade 1. Globally, EIV outbreaks (Florida clade 1) have been recently reported in the USA and South America (Argentina, Colombia, Chile, Ecuador, and Uruguay) (OIE 2019a). Many reports of EIV outbreaks have been reported worldwide. Mena et al. (2018) identified EIV of Florida clade 1 in outbreaks in Chile in January 2018 [28], followed by reports by Olguin-Perglione et al. (2019) and Castro et al. (2019) in Argentina and Uruguay, respectively [29,30]. Similar outbreaks were also reported in Europe, including Belgium, Denmark, France, Ireland, the Netherlands, Sweden, and the UK [31,32]. Recently EIVs identified in Senegal and Nigeria showed higher sequence identities to EIVs from South America than from Europe or the USA, suggesting an epidemiological link between West Africa and South America [33]. Similarly, outbreaks of FC1 in Asia were found to be introduced from the Americas, as reported in Argentina to Dubai, North America to Japan, and North America to Malaysia [34]. These findings reflect the impact of the global transport of horses on the spread of the H3N8 strains.

In the current study, the full-length genome analysis of the Saudi H3N8 strains revealed a low rate of mutations in different segments of the virus. This finding agrees with other findings and confirmed the fact that influenza viruses from mammals showed a slow rate of evolution [35]. This fact could be related to a weak immune selection or to host-related factors. Weak immune selection was suggested to be one of the reasons that EIV had a reduced rate of mutations [12]. In contrast, non-significant variations of *d_N_*/*d_S_* ratios were found between the EIV evolution and those of swine-origin H1N1/09 and human H3N2 influenza A viruses [36,37]. Accordingly, the detected low mutation rate in EIV needs further investigation.

Although vaccination is the most useful prophylactic strategy; continuous genetic evolution of the virus demands genetic characterization of currently circulating EIVs for the selection of a candidate vaccine strain. As vaccine failures have occurred in several parts of the world, there is a need for a better vaccine to completely eradicate equine influenza [38]. Hence, understanding of the molecular mechanisms involved with its cross-species transmission is of prime importance to devise any prophylactic and control strategy [39]. Interestingly, in Saudi Arabia, both Calvenza 03 (Florida clade 1: A/equine/Ohio/2003, American clade: A/equineKentucky/2/95, and Eurasian clade:A/equine/Newmarket/2/93) and Fluvac Innovator EHV-4/1 (Equine rhinopneumonitis types 1 and 4 viruses as well as A/equine-2/Kentucky/97) are currently approved for use to vaccinate horses against EIV. The former multivalent EIV contains A/equine/Ohio/2003, which showed the highest identity (only 10 amino acid substitutions in HA1 the Saudi strains) to the Saudi strains, while the latter vaccine showed lower identity (only 16 amino acid substitutions in HA1 the Saudi strains) to the Saudi strains. In addition, the OIE expert surveillance panel recommended the inclusion of both Florida clade 1 and 2 in EIV vaccines [30].

## 5. Conclusions

There are around 39,000 horses in Saudi Arabia, with some used in races or horse shows travelling across borders. Routine surveillance systems for emerging EIV strains are needed. Meanwhile, re-evaluation of the currently approved vaccines for EIV is recommended based on both the strain variation and latest recommendations of OIE. Importation of infected horses could be among the possible causes. A vaccination certificate is needed to confirm that animals have received at least one dose of the vaccine. Another booster 4 to 6 weeks before importing, exporting, or hosting horses is highly recommended to prevent the re-introduction of the EIV into Saudi Arabia.

## Figures and Tables

**Table 1:** Screening of equine influenza type A virus using real time PCR among horses from different geographical regions in Saudi Arabia.

Region	Number of Barns ^1^	Total Number of Animals	Animals Showing Clinical Signs n (%)	Mortality Rate Number (%)	Fatality Rate (%)	No. of Positive Pool Samples from the Examined Pools ^2^
Eastern region	30	272	78 (28.7)	1 (0.37)	1.28	21/30
Riyadh	10	158	33 (20.9)	0 (0.0)	0	7/10
Makkah	5	70	65 (92.9)	0 (0.0)	0	3/5
Al-Madinah	8	100	30 (30)	0 (0.0)	0	5/8
Al-Qassim	2	12	9 (75)	0 (0.0)	0	1/2
Tabuk	2	9	9 (100)	0 (0.0)		2/2
Total	57	621	224 (36.1)	1 (1.0)	0.45	39/57

^1^ The horse barns in different geographical regions were screened. The total number of the horses in each barn was recorded. ^2^ Nasal swabs from horses showing respiratory distress were collected individually, then pooled into small pools. Each pool was screened using quantitative reverse transcriptase real time polymerase chain reaction (qRT-PCR) targeting the matrix gene of the influenza A virus.

**Table 2:** Amino acid substitutions of the all the genomic segments of the Saudi equine H3N8 strains in comparison to the closely related strains.

	PB2		PB1	PA		HA		NP	NA	
H3N8 Strain	27	482	149	90	460	163	372	2	74	75
Equus ferus caballus/USA/51053/2019	H	K	I	I	I	T	A	K	I	D
A/Equuscaballus/USA/149632/2018	H	K	V	V	M	T	A	K	M	E
A/horse/Saudi Arabia/CVRL-193/2019	Q	K	V	V	M	I	T	K	M	E
A/horse/Saudi Arabia/CVRL-197/2019	H	K	V	V	M	I	T	E	M	E
A/horse/Saudi Arabia/CVRL-Ali/2019	H	T	V	V	M	I	T	K	M	E
A/donkey/Nigeria/VRD19 ZM20 T10 19RS459-5/2019	H	K	V	- *	- *	T	A	K	I	E
A/equine/Malaysia/1/2015	H	K	V	V	M	T	A	K	I	E

Both M and NS segments were not included in the comparison since they showed identical amino acid sequences to the Equus ferus caballus/USA/51053/2019. * Dashes mean sequences are not available.

## Data Availability

The data presented in this study are available in the article and in a NCBI database. Accession numbers are mentioned in the current article.
